# Direct embryonic biopsy with transcervical embryoscopy is an effective method for karyotyping and morphology assessment in miscarriages

**DOI:** 10.1007/s10815-024-03134-5

**Published:** 2024-05-17

**Authors:** Pinar Calis, Bengisu Akcay, Gurkan Bozdag, Mehmet Erdem, Ahmet Erdem, Meral Yirmibes, Esra Tug, Deniz Karcaaltincaba

**Affiliations:** 1 Department of Obstetrics and Gynecology, Gazi University Faculty of Medicine, Besevler, Ankara, 06650 Turkey; 2 Bahceci Health Group, Istanbul, Turkey; 3 Department of Medical Genetics, Gazi University Faculty of Medicine, Ankara, Turkey

**Keywords:** Miscarriage, Transcervical embryoscopy, Karyotype, Morphology, Embryo

## Abstract

**Purpose:**

The main purpose of this study is to compare the validity of transcervical embryoscopy method with standard uterine evacuation method in detecting more accurate karyotypes in miscarriages below tenth week of pregnancy. Additionally, the frequency and distribution of fetal morphological abnormality were evaluated.

**Methods:**

A prospective study was carried out at the Gazi University Faculty of Medicine, Department of Obstetrics and Gynecology. Patients with missed abortions between sixth and tenth gestational weeks were included in the study group, and fetal morphological examination and direct embryonic biopsy were performed by transcervical embryoscopy. The control group consisted of patients who experienced miscarriage and genetic material obtained from routine uterine evacuation between February and October 2023.

**Result:**

A total of 60 patients in the study group and 189 patients in the control group were evaluated. The median ages, previous miscarriage numbers, median gravida numbers, and median gestational weeks were comparable between groups. Chromosomal abnormality was detected in 24 (42.8%) and 52 embryos (29.9%) in the study and control groups, respectively (*p* = 0.004). Culture failure rates were 6.6% (*n* = 4) and 7.9% (*n* = 15) in the study and control groups, respectively. In the study group, 12 embryos had a morphological abnormality in which 6 of them had normal karyotype.

**Conclusion:**

Direct embryonic biopsy with transcervical embryoscopy is an effective method to exclude maternal decidual cell contamination and placental mosaicism in miscarriages for karyotype analysis. In addition, detecting anomalies in morphology might contribute our understanding in the process of miscarriages which arises independent from structural/numerical chromosomal abnormalities.

## Introduction

Miscarriage, according to the European Society of Human Reproduction (ESHRE), is defined as the spontaneous demise of pregnancy before 24 weeks of gestation [1]. Approximately 15–25% of all known pregnancies result in miscarriage, which mainly occurs before the 10th gestational week [2, 3]. Nevertheless, up to 50% of women experience at least one miscarriage during their reproductive lifespan. Although the most detectable reason for spontaneous miscarriage is a chromosomal abnormality in the embryo [2, 4, 5], maternal obesity, smoking, uterine congenital malformations, thyroid dysfunction, or chronic endometritis might also be encountered [6].

When numerical or structural chromosomal abnormalities are excluded in the fetus, it is noteworthy to emphasize that significant fetal morphological anomalies might still be potential factors for miscarriages. According to a study by Visconti et al., patients with previous fetal malformations had a four-times higher risk of recurrent miscarriage [7]. The most frequent anomalies were the central nervous system, the urogenital tract, and bone malformations. However, the exact frequency has not been reported, given that all gross anomalies cannot be detected with ultrasonography within the first trimester.

In the ESHRE guideline (2023), it was recommended that genetic analysis of pregnancy tissue following pregnancy loss, although not routinely considered, could be performed for explanatory purposes [1]. If desired, genetic analysis is performed using tissue acquired from uterine evacuation. However, curettage material contains fetal and maternal tissues and has a high maternal decidual cell contamination (MCC) ratio. To rule out MCC, microsatellite analysis (MSA), short-tandem-repeat (STR), or single-nucleotide-polymorphism (SNP) techniques can be performed. To confirm or exclude the occurrence of misleading male tissue in miscarriage material with XX karyotype, polymerase chain reaction amplification of the sex determination of Y chromosome (SRY) gene and/or Y-linked loci by interphase fluorescence in situ hybridization (FISH) with X/Y-specific DNA probes can be performed [8, 9]. ESHRE guideline (2023) strongly recommends performing array-comparative genomic hybridization (array-CGH) due to the reduced effect of MCC. However, a significant disadvantage of these methods is the high financial burden and the complexity of the technical steps. Apart from that, more than half of early spontaneous miscarriages contained no embryonic/fetal parts in materials sent for genetic examination [10, 11].

Transcervical embryoscopy is performed by passing through a rigid hysteroscope into the vaginal cavity, the cervical canal, and therefore the endometrial cavity, respectively, to visualize missed fetus. Hence, this technique allows direct visualization of a missed embryo in utero by not damaging the embryo. In 2003, Philipp et al. performed transcervical embryoscopy on miscarriages and obtained chorionic biopsies for karyotype analysis. According to the study, 75% of the miscarriages had abnormal karyotypes, and 18% had morphological abnormalities with normal karyotypes. The authors reported that fetal morphology could be visualized and abnormalities resulting in miscarriage could be detected [10].

The aim of this study is to compare the priorities of two methods (standard uterine evacuation method and transcervical embryoscopy method) to detect the correct karyotype of miscarriages occurring before the tenth week of gestation and to share preliminary results. In addition, we also evaluated the frequency and distribution of fetal morphologic abnormalities under direct visualization using embryoscopy.

## Materials and methods

The current prospective study was conducted after institutional ethics committee approval (Approval No.: 745) at Gazi University Faculty of Medicine (Ankara, Turkey) and included first-trimester miscarriage patients who underwent routine uterine evacuation or transcervical embryoscopy between February and October 2023. The patients were invited to the study if a fetal pole was detected in transvaginal ultrasonography Voluson E10 (GE Healthcare, Istanbul, Turkey) without a fetal heartbeat and sixth to tenth gestational week according to crown-rump length (CRL) measurement in ultrasound (3.58–30.8 mm). Transcervical embryoscopy was planned if the patient gave informed consent.

**Fig. 1:**
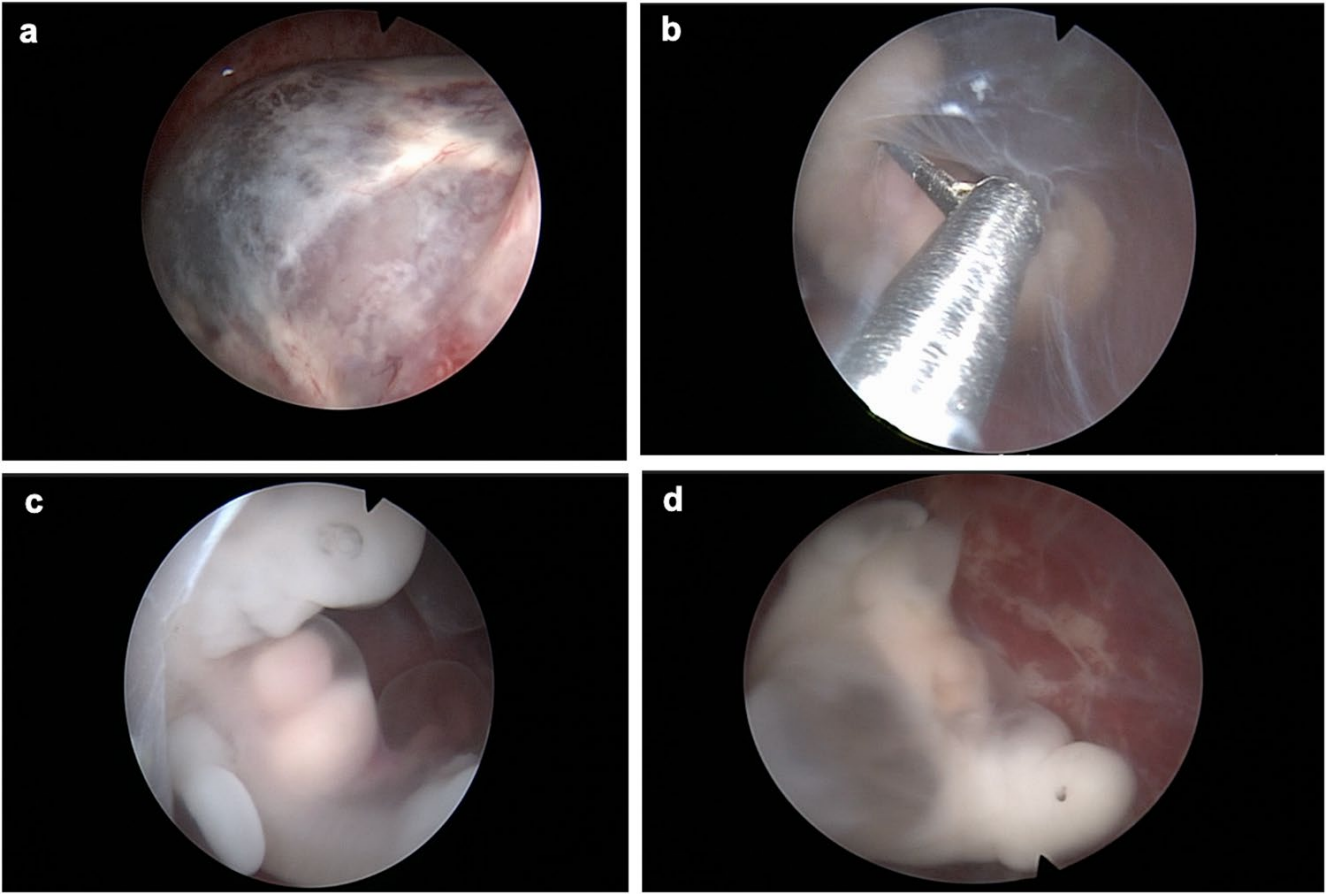
**a** Visualizing a gestational sac in the utero cavity. **b** Visualizing an amniotic sac and an embryo, with a micro scissor cutting the amniotic sac to see the embryo better. **c** Exploring a normal morphology embryo. **d** Exploring an embryo with cystic hygroma

Transcervical embryoscopy was performed with laryngeal mask airway (LMA) in a dorsal lithotomy position. The rigid hysteroscopy with a 30-degree angle view with both the biopsy and irrigation working channels (Circon Ch 4.8 mm, Storz, Tuttlingen, Germany) was inserted into the intrauterine cavity. Except for the visualization of the embryo, continuous normal saline flow (pressure ranging from 40 to 120 mm Hg) was used throughout the procedure. During visualization of the embryo, the saline flow was stopped in order not to disrupt the embryo. After visualizing the chorionic sac, by a micro scissor of the hysteroscopy, the chorionic sac and then the amniotic sac were cut, and a hysteroscope was inserted into the amniotic sac. The embryo and yolk sac of the pregnancy were observed and systematically evaluated in the following order: head, face, spine, abdominal wall, and upper and lower extremities, if possible. The concordance of the observed embryo size with the estimated gestational week according to the last menstrual period was also assessed (Fig. 1). Transcervical embryoscopy was performed by a single surgeon (P.C).

The study group consisted of participants who underwent transcervical embryoscopy. For this procedure, the grasper of the hysteroscopy was used to sample the embryonic tissue through the uterine cavity. This tiny non-united embryonic tissue was placed in the transport tube containing antibiotics and cell culture medium and quickly transported to the genetics laboratory for long-term tissue culture. Subsequent curettage was performed for the rest of the pregnancy material after the embryoscopy.

In the genetics laboratory, the embryonic tissue, consisting of blood clots, membranes, and solid tissues, was first separated from the blood in a sterile class II laminar cabinet. The remaining material was dissected in the same laminar cabin, and after several mechanical washings, two separate cell cultures were set up. The embryonic tissues were incubated at 37 °C and 5% CO_2_ using 25 cm_2_ culture dishes, including 0.75 mL of 20 mM l-glutamine, 0.5 mL penicillin/streptomycin (10,000 U/mL–100 mg/mL), and 3 mL medium supplemented with non-essential amino acids (BIOAMF-2 Medium-Biological Industries, Israel). After approximately 10 to 15 days of cultivation, a sufficient number of colonies were visible under an inverted microscope and 300 μL of Colcemid (0.010 mg/mL, Biological Industries, Israel) was added to the culture medium to arrest the spindles. After waiting for half an hour or 45 min, it was centrifuged; after discarding the supernatant, it was suspended in 10 mL of hypotonic solution (0.075 mol/1 KCl) for 35 min at 37 °C. The sample was treated 3–4 times with the fixative solution prepared with 1: 3 acetic acid:methanol. The cells were spread on slides and stained by the Giemsa-Trypsin banding method. A total of 10 to 20 metaphases derived from two primary cultures were analyzed under light microscopy for each sample, and the results were reported according to the International System for Human Cytogenetic Nomenclature (ISCN) 2020.

The control group consists of patients who had a miscarriage in the same period as the study group (February–October 2023) and at a similar gestational age, and whose genetic material was obtained from uterine evacuation. After vaginal lavage with povidone-iodine for uterine evacuation, a number six Karman cannula was placed through the cervical external os and all pregnancy material was aspirated under transabdominal ultrasound. The material was separated under the naked eye and placed in a transport medium with antibiotic for karyotype analysis. In the genetics laboratory, in a sterile class II laminar cabinet, the villous components of the trophoblastic material were carefully dissected to exclude other components of the material, such as blood clots. The tissue was washed two or three times with medium and placed in two separate flasks. The harvesting, staining, and analysis steps were repeated in the same way as for the materials obtained from the transcervical embryoscopy.

A total of 68 patients approved the study protocol. However, clear visualization could not be performed in 8 patients due to non-life-threatening hemorrhage in the uterine cavity or maceration of conception material for the length of time that had passed. Finally, transcervical embryoscopy was completed in 60 patients, and outcome parameters were compared with 189 control patients.

### Statistical analysis

Statistical analyses were performed using an SPSS v25.0 (IBM SPSS Inc., Chicago, IL) to compare karyotype abnormalities across the groups. Descriptive statistics were given by mean ± standard deviation (SD) and median (minimum–maximum). Karyotype abnormalities between the groups were compared using the chi-square test. A significance value was set at 0.05.

## Results

The median ages of the patients in the transcervical hysteroscopy and uterine evacuation groups were 32 (min–max, 19–43) and 31 (min–max, 19–44) years, respectively (*p* = 0.08). The respective median gravida numbers were 2 (min–max, 1–7) and 1 (min–max, 1–3), respectively (*p* = 0.93). The median miscarriage numbers were 2 (min–max, 1–6) and 2 (min–max, 1–3) in the study and control groups, respectively (*p* = 0.33). The median gestational weeks were 7 weeks and 2 days (min–max, 6 weeks 1 day–9 weeks 5 days) and 7 weeks and 5 days (min–max, 5 weeks 4 day–9 weeks 5 days) in the study and control groups, respectively (*p* = 0.83) (Table 1).

**Table 1:** Demographic values and their statistical difference between transcervical embryoscopy and control groups

	Transcervical embryoscopy	Control	*p* value
Age (years)	32 (19–43)	31 (19–44)	0.08
Gravida	2 (1–7)	1 (1–3)	0.93
No of previous miscarriage	2 (1–6)	2 (1–3)	0.33
Gestational age	7 weeks 2 days (6 weeks 1 day–9 weeks 5 days)	7 weeks 5 days (5 weeks 4 day–9 weeks 5 days)	0.83

*All values were given as median (min–max)*

In the transcervical embryoscopy group, 4 patients (6.6%) had culture failure. Of the remaining 56 patients, 24 embryos (42.8%) had a chromosomal abnormality, 32 embryos had a normal karyotype, and 12 had a morphological abnormality (21.4%) (Table 2). On the other hand, for the uterine evacuation group, 15 patients (~ %8) had culture failure. There was no statistically significant difference between the two groups (*p* = 0.12). Of the remaining 174 patients, chromosomal abnormalities were detected in 52 embryos (29.9%). There was a strong statistically significant difference in detecting chromosomal abnormality between the uterine evacuation and the transcervical embryoscopy approaches (*p* = 0.004).

**Table 2:** Type and number of karyotype abnormalities in transcervical embryo biopsy and control groups

	Transcervical embryoscopy (*n*)	Control (*n*)
Distribution of abnormal karyotype		
Trisomy 14		1
Trisomy 15		2
Trisomy 16	6	8
Trisomy 2		4
Trisomy 20		1
Trisomy 21		2
Trisomy 22	4	3
Trisomy 13	2	1
Trisomy 15	3	2
Trisomy 18		1
Trisomy 7		2
Trisomy 9		1
Trisomy 15 + trisomy 22		1
Trisomy 21 + trisomy 18		1
69,XXX + trisomy 7		1
69,XXY	1	4
69,XXX		4
92,XXXX		2
92 XXYY		1
Mosaic monosomy X		1
Monosomy X	8	7
46 XX t(1;13) (q42;q14)		1
Rob translocation trisomy 14		1
Subtotal	**24**	**52**
Distribution of normal karyotypes		
46 XX (*n*, %)	20 (62%)	102 (84%)
46 XY (*n*, %)	12 (38%)	20 (16%)
Subtotal	**32**	**122**
Total	**56**	**174**

Twelve embryos were detected as abnormal in morphological assessment in 60 patients with successful visualization with transcervical embryoscopy. Neural tube defect (*n* = 4), cystic hygroma (*n* = 2), abdominal wall defect (*n* = 4), and cranial defect (*n* = 2) were noted. Six of the 12 embryos with gross morphologic abnormality did not have chromosomal abnormalities, and 1 had culture failure.

## Discussion

This study proposes that the transcervical embryoscopy approach is superior to the conventional uterine evacuation approach in the detection of numerical karyotype abnormalities leading to miscarriage. The significant difference in the power of detecting genetic abnormalities using transcervical embryoscopy is related to the opportunity to identify and retrieve the embryo per se rather than to evacuate a mixture of maternal and fetal tissues. In addition, although lower culture failure rates were observed in the transcervical embryoscopy group, the result was not statistically significant. Notably, detecting abnormality in morphology might also be an important finding, particularly in pregnancies with normal karyotypes.

Utilization of transcervical embryoscopy in miscarried pregnancies was initially defined by Philipp et al. in 2001 [12] when (*n* = 19) patients were evaluated with transcervical embryoscopy, and simultaneous chorion biopsies were taken for karyotype analysis. In 10 out of 19 cases, multiple morphologic abnormalities were reported. For the detection of numerical chromosomal abnormalities in conception material, several studies have been reported evaluating the efficiency of transcervical embryoscopy while aiming to minimize the MCC rate and directly sampling from the chorion. To compare karyotype abnormalities in missed miscarriages, transcervical embryoscopy was performed to biopsy both the chorion and the embryo [13]. In that study, the difference in chromosomal abnormality between the chorion and the embryo could not be detected when biopsies were taken separately. In contrast, the authors noticed discrepancies in 3 cases in another study evaluating a total of 71 samples of conception material [14]. In addition, in a study by Philipp et al., obtaining chorionic biopsies with transcervical embryoscopy, 75% of miscarriages had abnormal karyotypes. The karyotype abnormality ratio was higher than the literature on miscarriages, which could be a result of chorionic tissue. However, due to its mosaicism, it cannot reflect the embryo karyotype 100% [10]. In light of the aforementioned studies, in our research, we preferred to retrieve a biopsy from the embryo directly to eliminate placental mosaicism. In the current study, our karyotype abnormality was 42.8% in the transcervical embryoscopy group; however, in the control group, it was only 29.9%. The lower ratio of chromosomal abnormality in the controls might be explained by two folds. Firstly, our study cohort is relatively young with a median age of 31. Nevertheless, as documented by a study including 15,169 consecutive trophectoderm biopsies evaluated with comprehensive chromosomal screening, the aneuploidy rate is around 30% [15]. Secondly, as the main hypothesis of the current study is based on, the evacuation of maternal, embryonic, and chorionic tissues might hamper the detection of genuine ploidy status. It was assumed that the reason for the significant difference between these rates was the high rate of maternal decidual cell contamination in the control group; karyotype abnormality was less than both the transcervical embryoscopy group and the literature. In both methods (transcervical embryoscopy and studies in literature), maternal decidual cell contamination was eliminated, and only missed embryo was analyzed for karyotype. If the sex ratio was analyzed between the control group and the study group, XX ratio was much higher in the control group than in the study group. This also shows MCC in the control group. Array-CGH can be used as an alternative method to detect chromosomal gains and losses in aborted material, but the inability to detect low mosaic cases and high funding are limitations of this technique. The priority of conventional karyotyping after the transcervical embryoscopy technique is not only to reduce the MCC rate but also to provide the opportunity to detect low-level mosaic karyotypes compared to array-CGH. Another important point of the study is that the morphological abnormality of the embryo can be directly evaluated during embryoscopy.

A limited number of studies, similar to ours, performed transcervical embryoscopy for both karyotype analysis and morphology assessment. In the study by Feichtinger et al., as expected, aneuploidy was found to be strongly associated with morphological defects, but interestingly, among patients with secondary pregnancy loss (three or more consecutive pregnancy losses after a successful pregnancy) with a euploid karyotype, 55% had embryos that also exhibited morphological defects [16]. A study by Philipp et al. involved karyotype analysis and morphological evaluation in 233 missed abortions by transcervical embryoscopy. Contrary to the previous research, 18% had a normal karyotype with morphological defects. However, all the studies in the literature were based on karyotype sampling from chorion biopsies. In our study, direct embryo biopsies were taken for the karyotype analysis, and morphological evaluation was also conducted. From this point, this is a pioneer study in the literature that involves both karyotype and morphological analysis in early missed abortions by obtaining direct embryo biopsies.

One might claim that direct biopsy from the embryo might obtain small amount of material when compared with chorion biopsy and hence would fail to produce a result following tissue culture [14]. Nevertheless, taking biopsy from the chorion has been more frequently evaluated among the studies possibly due to its technically easiness [10, 16]. However, according to our data, we did not notice more frequent failure after culture when biopsy has been directly taken from the embryo. This discrepancy might be related with two points. Firstly, the duration between the notification of missed miscarriage and transcervical hysteroscopy might be distinct in our study and previous studies, which has not been documented in any of the manuscripts, unfortunately. Secondly, the gestational week of the fetal demise might contribute to the results, as already referred by Ferro et al. (2003) previously. Therefore, the optimal duration after demise and weeks of gestation should be distinguished among all cases which would benefit most with transcervical hysteroscopy in further trials with larger sample size.

The most challenging aspect of these techniques is to obtain an embryonic biopsy without disrupting the embryo while extracting it from the uterine cavity, as it is fragile. For karyotype assessment, the material was always adequate. Apart from this, in some cases, while embryo size was insufficient to evaluate morphology, all samples could be sent for karyotype analysis. In particular, in patients with hemorrhage in the uterine cavity or maceration of conception material due to a long period passing after miscarriage, fetal visualization was inadequate, so those patients were not appropriate for transcervical embryoscopic evaluation. Since the same situation is a factor that negatively affects cell culture and causes microorganism contamination, results could not be achieved in a small number of samples due to culture failure. Additionally, by transcervical embryoscopy, the defects in visceral organs cannot be detected. From this point of view, the estimated rate of morphological abnormalities, regardless of karyotype, may be even higher. These undetectable abnormalities can be one of the reasons for early missed abortions with normal karyotypes and can potentially be the explanation for the idiopathic portion. In addition to that, in conventional approach to eliminate MCC, after uterine evacuation, array-CGH should be applied. Although the cost of the procedure might change in distinct settings, government-based social insurance refers to a cost of 15 USD for hysteroscopy but corresponds to 110 USD for uterine evacuation and array-CGH (https://www.saglikaktuel.com/mobi/haber/25-subat-2024-degisiklik-tebligleri-islenmis-guncel-2013-sut-sgk-95861.htm). Therefore, in our setting, transcervical embryoscopy seems to be less costly. Plus, to find the possible hidden Y chromosome component of trophoblastic tissues with XX karyotype, the sex-determining region of the Y chromosome (SRY) gene can be investigated by polymerase chain reaction or by using fluorescence in situ hybridization (FISH), and the centromeric region of chromosome Y can be stained on interphase/metaphase derived from trophoblastic tissues. We would like to perform PCR amplification of SRY in these control samples and/or search for Y-linked loci by interphase fluorescence in situ hybridization (I-FISH) with X/Y-specific DNA probes, also to detect 45,X karyotype ratio, but unfortunately, we have no remaining samples (fixative liquid containing intermediate phases and/or DNA samples). The second point is that it might not be feasible and cost-effective to perform SRY gene amplification and/or FISH analysis in routine laboratory practices.

In conclusion, transcervical embryoscopy effectively rules out maternal decidual cell contamination in miscarriages for karyotype analysis. Direct embryonic biopsy is more trustworthy than chorionic biopsy by excluding placental mosaicism. Apart from detecting any chromosomal abnormality, using transcervical embryoscopy allows for visualization of fetus morphology and gives a chance to diagnose potential reasons for miscarriage, which are missed when using a conventional approach. Detecting morphological abnormality and performing karyotype analysis using this approach may be a suitable method for selecting and cytogenetically evaluating embryos before further genetic evaluations such as microarray or whole-genome sequencing. Other studies with a more extensive patient series would be beneficial for definitive conclusions.

## Data Availability

The data that support the findings of this study are available on request from the corresponding author (PC).
